# The protein-protein interaction between two spliced leader *trans* -splicing factors is mediated by two interlinked α-helical domains.

**DOI:** 10.17912/micropub.biology.001705

**Published:** 2025-10-22

**Authors:** Peter Eijlers, Jonathan Pettitt, Berndt Müller

**Affiliations:** 1 School of Medicine, Medical Sciences and Nutrition, University of Aberdeen, Aberdeen, Scotland, United Kingdom

## Abstract

SNA-1 and SNA-2 proteins are involved in spliced leader *trans* -splicing in *Caenorhabditis elegans* . They are components of the SL1 snRNP that donates the spliced leader 1 RNA which replaces the 5' end of most pre-mRNAs. SNA-1 and SNA-2 bind to each other, but the nature of their interaction is unclear. AlphaFold-Multimer predicts that the central region of SNA-1 and the C-terminal region of SNA-2 , each consisting of 3 α-helices, interlink to bring about the interaction between these proteins. Using yeast 2-hybrid assays we demonstrate that these regions are required and sufficient for this unusual mode of interaction between two proteins.

**Figure 1. Identification of domains necessary and sufficient for the interaction between C. elegans SNA-1 and SNA-2 proteins:**
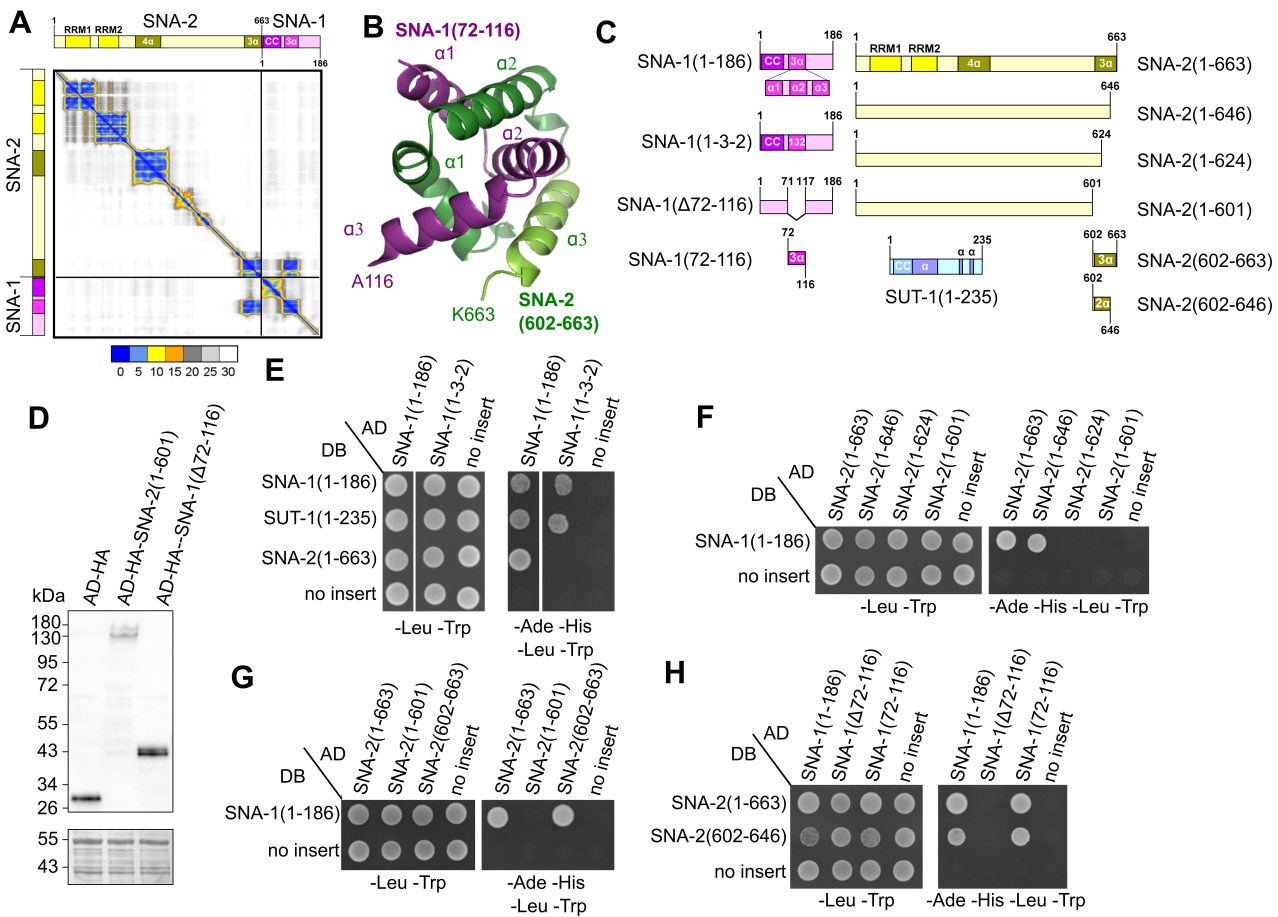
(A) Predicted alignment error heat map showing the predicted error (in Angstroms) between all pairs of residues for the structure of the SNA-2 / SNA-1 complex modelled using AlphaFold-Multimer. Interacting domains are identified in the bottom left and top right rectangles. The bar indicates the error colour scheme. The schematic representations of SNA-2 and SNA-1 protein indicate the position of protein domains and identify regions involved in the interaction between SNA-1 and SNA-2 . CC, coiled coil; RRM, RNA recognition motif; 4α, domain with 4 antiparallel α-helices; 3α, domain with 3 α-helices. (B) Cartoon representation of the AlphaFold-Multimer predicted interaction between SNA-1 and SNA-2 proteins. Shown are SNA-2 amino acids S602-K663 and SNA-1 amino acids S72-A116. (C) Schematic representation of proteins expressed either as Gal4 DNA-binding domain fusion proteins (DB) from derivatives of pGBKT7 in Y2HGold yeast, or as Gal4 activation domain fusion proteins (AD) from derivatives of pGADT7 in Y187 yeast. SNA-1 (1-186), SNA2(1-663) and SUT-1 (1-235) are the full-length proteins and the various truncations and deletions are indicated. (D) Western blot confirming that SNA-1 (∆72-116) and SNA-2 (1-601) proteins are expressed. Shown are extracts prepared from diploid yeast carrying pGADT7, pGADT7- SNA-2 (1-601) or pGADT7- SNA-1 (∆72-116) expressing HA-tagged activation domain only or fused to SNA-2 (1-601) or SNA-1 (∆72-116) proteins, respectively. Proteins were detected by probing with anti-HA antibodies (top panel) and using amido black to demonstrate equal loading (bottom panel). (E, F, G, H): Diploid yeast expressing the indicated Gal4 activation domain (AD) and Gal4 DNA-binding domain (DB) fusion proteins were grown as control on synthetically defined (SD) medium lacking leucine and tryptophan (-Leu -Trp) and on SD medium lacking adenine, histidine, leucine and tryptophan (-Ade -His -Leu -Trp) to test for protein-protein interaction. Control diploids (no insert) carry either pGADT7 expressing the activation domain only or pGBKT7 expressing the DNA binding domain in combination with indicated second plasmids. (E) Swapping the order of SNA-1 α-helices α2 and α3 located between amino acids G86 and K115 abolishes the interaction with SNA-2 , but not with SUT-1 or SNA-1 . (F) Truncation of the SNA-2 C-terminus abolishes interaction with SNA-1 . (G) The C-terminal SNA-2 fragment SNA-2 (602-663) is sufficient for the interaction with SNA-1 . (H) SNA-1 amino acid region 72-116 is required ( SNA-1 (∆72-116)) and sufficient ( SNA-1 (72-116)) for the interaction with full-length SNA-2 and the SNA-2 fragment SNA-2 (602-646).

## Description

Spliced leader *trans* -splicing is an essential mRNA processing step that occurs in many eukaryotes. It produces the mRNA 5' end by replacing the majority of the genomically encoded 5' untranslated region of pre-mRNA with a short RNA, the spliced leader, which is encoded elsewhere in the genome. This reaction is mechanistically analogous to the removal of introns during pre-mRNA splicing (Blumenthal 2012; Pettitt et al. 2010). Spliced leader *trans* -splicing requires spliceosome components and a spliced leader snRNP (SL snRNP) that contains spliced leader RNA (SL RNA), the precursor RNA that donates the spliced leader. In *C. elegans ,* the spliced leader 1 (SL1) RNA sequence constitutes the 5' end of most pre-mRNAs and is donated by the SL1 snRNP. The proteins SNA-1 , SNA-2 and SUT-1 are involved in spliced leader *trans* -splicing (MacMorris et al. 2007; Philippe et al. 2017). SNA-1 and SNA-2 are part of the SL1 snRNP (MacMorris et al. 2007; Fasimoye et al. 2022; Eijlers et al. 2024). SUT-1 protein associates with SmY snRNPs that are thought to be involved in the recruitment of the SL1 snRNP to pre-mRNA for the initiation of spliced leader *trans* -splicing (Eijlers et al. 2024).

The molecular structures of SNA-1 (Uniprot O45149), SNA-2 (Uniprot Q94050) and SUT-1 proteins (Uniprot A9D649) have been predicted by AlphaFold (Jumper et al. 2021). The main structural features of SNA-2 are two domains with RNA recognition motifs (RRM1, RRM2) between amino acids 33 and 233 followed by a domain with a bundle of 4 anti-parallel α-helices between amino acids 260-341 and a domain with 3 α-helices at the C-terminus between amino acids 607-662 ( Figure 1A, 1C). Between these two domains, there are additional α-helices and β-strands that are not part of distinct protein domains. These features are for clarity's sake not included in the diagrams in Figure 1. SNA-1 is predicted to contain two antiparallel α-helices forming a coiled-coil structure at the N-terminus between amino acids 4-61 followed by a region containing 3 α-helices between amino acids 73-116 ( Figure 1A, 1C). SUT-1 is predicted to have a coiled-coil structure formed by two antiparallel α-helices at the N-terminus between amino acids 10-58, with the second α-helix extending for an additional 64 amino acids ( Figure 1C ). This is followed by a 113 amino acid long region containing 3 β-strands (not included in Figure 1C ) and two short α-helices.

We have shown earlier that SNA-1 and SNA-2 proteins interact with each other, and have ruled out that the SNA-2 RRMs and the SNA-1 coiled-coil region are involved in the formation of this complex (Fasimoye et al. 2022). We also found that SNA-1 interacts with SUT-1 , and with itself (Fasimoye et al. 2022).

Here we used the AlphaFold-Multimer tool (Evans et al. 2021) to further investigate the interaction between SNA-1 and SNA-2 . Figure 1A shows the predicted alignment error of the modelled protein complex structure. The SNA-2 and SNA-1 domains introduced above have a low predicted alignment error, indicating that there is high confidence in the secondary and tertiary structures of these domains. This analysis also predicts that the interaction between SNA-2 and SNA-1 is brought about by the interlinking of the 3 α-helix region at the SNA-2 C-terminus (between residues P607 and K663) with the central region of SNA-1 (between residues S72 and A116) that also contains 3 α-helices ( Figure 1A, 1B). AlphaFold-Multimer did not make any high confidence predictions for regions involved in SNA-1 homomeric or SNA-1 / SUT-1 heteromeric interactions.

To confirm that the domains identified using AlphaFold-Multimer are indeed involved in the interaction between SNA-1 and SNA-2 , we exploited yeast 2-hybrid assays to detect the interaction between these two proteins (Fasimoye et al. 2022). To test the role of the putative protein regions involved in the SNA-1 / SNA-2 interaction we created derivatives by site-directed mutagenesis of yeast 2-hybrid vectors expressing SNA-1 or SNA-2 proteins ( Figure 1C ).

We first tested whether the protein sequence of the central region of SNA-1 containing the 3 α-helices is required for the interaction with SNA-2 . As expected, wild-type SNA-1 (1-186) interacted with full-length SNA-2 (1-663), SNA-1 (1-186) and SUT-1 (1-235) ( Figure 1E ). However, a modified SNA-1 protein (designated “1-3-2”), in which the order of the second and third α-helices (α2: G86-S98, and α3: T102-K115, respectively) is switched, was unable to interact with full-length SNA-2 (1-663). In contrast, SNA-1 (1-3-2) interacted with full-length SUT-1 (1-235) and SNA-1 (1-186). This is compatible with the SNA-1 region between G86 and K115 (corresponding to α-helices 2 and 3) being involved in the interaction with SNA-2 , but that interactions between SNA-1 / SNA-1 and SNA1/SUT1 are mediated by other regions of SNA-1 . It also implies that not only the protein secondary structure, but also the amino acid sequence is important for this protein-protein interaction.

We then focused on the role of the SNA-2 C-terminus in protein-protein interaction between SNA-1 and SNA-2 . Removal of the C-terminal 17 amino acids to create SNA-2 (1-646) did not abolish the interaction with full-length SNA-1 (1-186) ( Figure 1F ). However, further truncation of the SNA-2 C-terminus ( SNA-2 (1-624) and SNA-2 (1-601)) prevented the interaction with SNA-1 (1-186). This loss of SNA-1 / SNA-2 protein interaction is not caused by truncation of the C-terminus eliminating SNA-2 protein expression ( Figure 1D ).

To determine whether the SNA-2 C-terminal region is sufficient for the interaction with SNA-1 , we prepared a construct that expresses SNA-2 (602-663) that lacks amino acids 1-601. This protein interacted with full-length SNA-1 (1-186) protein, whereas SNA-2 (1-601) lacking the C-terminal 62 amino acids showed no interaction ( Figure 1G ). Together, this indicates that the C-terminal region of SNA-2 is required and sufficient for the interaction with SNA-1 .

To further investigate the region of SNA-1 involved in the interaction with SNA-2 , we produced SNA-1 (∆72-116) expressing SNA-1 lacking amino acids 72 to 116, the region predicted to be involved in the interaction with SNA-2 . This protein was expressed ( Figure 1D ) but did not interact with full-length SNA-2 (1-663) protein ( Figure 1H ). On the other hand, SNA-1 (72- 116) protein spanning the 3 α-helices but lacking N- and C-terminal flanking sequences, interacted with full-length SNA-2 (1- 663) ( Figure 1H ). Together with the observation that swapping SNA-1 α-helices α2 and α3 abolished interaction with SNA-2 ( Figure 1E ), this indicates that the SNA-1 region between amino acids 72 and 116 is required and sufficient for the interaction with SNA-2 protein.

The SNA-2 region required for the interaction with SNA-1 identified ( Figure 1G ) was further refined by removing the C- terminal α-helix from SNA-2 (602-663) to produce SNA-2 (602-646). This protein interacted with full-length SNA-1 (1-186) and with SNA-1 (72-116), but not with SNA-1 (∆72-116) ( Figure 1H ).

In conclusion, AlphaFold-Multimer modelling predicted that a central α-helical region of SNA-1 and a C-terminal α-helical region of SNA-2 are the domains responsible for the interaction between these two proteins ( Figure 1A, 1B). Using yeast 2-hybrid assays we experimentally confirmed that these domains are required ( Figure 1E, 1F, 1H) and sufficient for SNA-1 / SNA-2 protein-protein interaction ( Figure 1G, 1H). Together, this indicates that these α-helical regions are *bona fide* protein-protein interaction domains.

To the best of our knowledge, this mode of protein-protein interaction is unusual. The modelling predicts that the protein-protein interaction between SNA-1 and SNA-2 is mediated by the interlinking of these α-helical domains ( Figure 1B ). A structurally similar interaction has been reported in homodimer formation of the *Helicobacter pylori* protein HP0242 (Tsai et al. 2006; King et al. 2010). This structure is reminiscent of trefoil knots found in natural and engineered proteins (Doyle et al. 2023; Jamroz et al. 2015), and it has been shown that a tandem repeat of the HP0242 protein folds to form a trefoil knot (King et al. 2010). It will be interesting to further investigate the molecular basis of the interaction between SNA-1 and SNA-2 .

SNA-1 and SNA-2 are components of the SL1 snRNP that donates the spliced leader 1 to pre-mRNA (MacMorris et al. 2007; Fasimoye et al. 2022; Eijlers et al. 2024). As SNA-2 is an essential function and *sna-1* mutation leads to cold-sensitive defects in viability, this interaction is likely critical for spliced leader *trans* -splicing (MacMorris et al. 2007, Philippe et al. 2017). The findings described here will inform an examination of the significance of the SNA-1 / SNA-2 interaction *in vivo.*

## Methods

**Molecular Cloning.** Constructs for yeast 2-hybrid assays expressing full-length SNA-1 (1-186), SNA-2 (1-663) and SUT-1 (1-235) from pGADT7 and pGBKT7 were described earlier (Fasimoye et al. 2022). Constructs expressing truncated proteins, protein domains or otherwise modified versions of these proteins were produced using the Q5 Site-Directed Mutagenesis Kit (New England Biolabs) and propagated in XL1-Blue *E. coli* . Plasmids and primers are listed in Tables 1 and 2, respectively. pGADT7-SNA1(∆72-116) expressing the GAL4 activation domain fused to SNA-1 lacking amino acids 72-116 was derived from pGADT7- SNA-1 (1-186) using primers SNA-1 (∆centr)F and R to delete the region coding for these amino acids. pGADT7- SNA-1 (1-3-2) expressing SNA-1 with α-helices 2 (G86-S98) and 3 (T102-K115) exchanged was derived from pGADT7- SNA-1 (1-186) using primers SNA-1 (132)F and R to replace the original coding sequence.

pGADT7- SNA-1 (72-116) expressing SNA-1 amino acids 72-116 was derived from pGADT7- SNA-1 (1-186) using primers SNA-1 (72-186)F and R to delete the coding region for amino acids 1-72, and then primers SNA-1 (72-116)F and R to delete coding region for amino acids 117-186, leaving the stop codon in place.

pGADT7- SNA-2 (1-663) derivatives pGADT7- SNA-2 (1-601), pGADT7- SNA-2 (1-624) and pGADT7- SNA-2 (1-646) were produced by introducing deletions into the SNA-2 coding region but leaving the original stop codon in place, using primers SNA-2 (∆Ct)F and R, SNA-2 (1-624)F and R, and SNA-2 (1-646)F and R, respectively.

pGADT7- SNA-2 (602-663) expressing the GAL4 activation domain fused to SNA-2 (602-663) was derived from pGADT7- SNA-2 (1-663) by deleting the SNA-2 coding region for amino acids 1-601 using primers SNA-2 (602-663) F and R. pGBKT7- SNA-2 (602-646) was derived from pGBKT7- SNA-2 (602-663) by deleting the coding region for amino acids 646-663 and leaving the stop codon in place, using primers SNA-2 (602-646)F and R. The plasmid sequences were confirmed by Sanger sequencing (Eurofins Genomics).

**Yeast 2-hybrid assays.** Yeast 2-hybrid assays were performed using standard protocols as described earlier (Fasimoye et al. 2022). Briefly, pGADT7 and derivatives were transformed into Y187 and pGBKT7 and derivatives were transformed into Y2HGold. After mating, diploids were grown on plates with synthetic defined medium lacking leucine and tryptophan (-Leu - Trp). For spot tests, diploids were grown overnight in liquid synthetic defined -Leu -Trp medium at 30⁰C. Cultures were then diluted to an OD600 of 0.1 in sterile water. 10 µL of each dilution were spotted onto plates with synthetic defined -Leu -Trp and plates with synthetic defined -Ade -His -Leu -Trp medium, and grown at 30⁰C for 2-3 days.

**Western blotting.** Protein was extracted from yeast diploids as described (von der Haar 2007). The equivalent of 3.6 x 10 ^6^ cells was separated by SDS-PAGE using a NuPAGE 4%-12% Bis-Tris gel (Invitrogen) with MOPS SDS (Invitrogen) as buffer system. Proteins were transferred onto Hybond-P membranes (Cytiva) by wet transfer using NUPAGE transfer buffer (Life Technologies). The membrane was probed with 1:1000 Anti-HA.11 Epitope Tag Antibody (clone 16B12, BioLegend) and 1:3000 Anti- mouse IgG, HRP-linked Antibody (#7076, Cell Signalling Technology) and visualised by chemiluminescence (Immobilon Forte Western HRP substrate, Millipore) using an iBright FL1000 visualiser (Invitrogen). The membrane was subsequently stained with amido black to control for protein content.

**Prediction of protein-protein interaction.** The prediction was done using SNA-1 (186) (UniprotKB O45149) and SNA-2 (1- 663) (UniprotKB Q94050) amino acid sequences running the Alphafold2 extension Alphafold-Multimer (versions 2.2 and 2.3)(Jumper et al. 2021; Evans et al. 2021) in Google Colab under relaxed conditions for 3 or 6 cycles.

**Software.** The cartoon representation of the interacting region was made with PyMOL (version 2.5.7). The predicted alignment error plot was made with ChimeraX (release 1.8) (Meng et al. 2023) using the AlphaFold Crystallographic Information file and JSON file. The protein diagrams were designed in IBS (release 1.03) (Liu et al. 2015).

## Reagents

**Table:** 

**Table 1. Plasmids**		
**Plasmid**	**Expressed Protein**	**Source/Reference**
pGBKT7	Gal4 DNA binding domain	Takara Bio
pGADT7	GAL4 activation domain	Takara Bio
pGBKT7- SNA-1 (1-186)	DB- SNA-1 (1-186)	(Fasimoye et al. 2022)
pGADT7- SNA-1 (1-186)	AD- SNA-1 (1-186)	(Fasimoye et al. 2022)
pGADT7- SNA-1 (Δ72-116)	AD- SNA-1 (1-71,117-186)	This study
pGADT7- SNA-1 (1-3-2)	AD- SNA-1 (1-3-2)	This study
pGADT7- SNA-1 (72-116)	AD- SNA-1 (72-116)	This study
pGADT7- SNA-2 (1-663)	AD- SNA-2 (1-663)	(Fasimoye et al. 2022)
pGADT7- SNA-2 (1-601)	AD- SNA-2 (1-601)	This study
pGADT7- SNA-2 (1-624)	AD- SNA-2 (1-624)	This study
pGADT7- SNA-2 (1-646)	AD- SNA-2 (1-646)	This study
pGADT7- SNA-2 (602-663)	AD- SNA-2 (602-663)	This study
pGBKT7- SNA-2 (1-663)	DB- SNA-2 (1-663)	(Fasimoye et al. 2022)
pGBKT7- SNA-2 (602-646)	DB- SNA-2 (602-646)	This study
pGBKT7-SUT1(1-235)	DB- SUT-1 (1-235)	(Fasimoye et al. 2022)

**Table:** 

**Table 2. Primers**		
**Name**	**Sequence**	**Plasmid**
SNA-1 (132)F	atcctggtgctggctttgcacaagcaattcaaattgcaaactccgctaatccaccacagcctgtatatc	pGADT7- SNA-1 (1-3-2)
SNA-1 (132)R	ctgctttaaaattgtgaaattcctgttgtaatgttggccaaaatgtatctactttatccagcaactctc	
SNA-1 (72-186)F	agcatcgatgcagtc	pGADT7- SNA-1 (72-186)
SNA-1 (72-186)R	gaattcactggcctc	
SNA-1 (72-116)F	taaggatccatcgagc	pGADT7- SNA-1 (72-116)
SNA-1 (72-116)R	tgctttaaaattgtgaaattcc	
SNA-1 (∆centr)F	aatccaccacagcctgtatat	pGADT7-SNA1(Δ72-116)
SNA-1 (∆centr)R	tttcgggcctgattcgac	
SNA-2 (∆Ct)F	taaggatccatcgagctc	pGADT7- SNA-2 (1-601)
SNA-2 (∆Ct)R	agccgttgtagtacgtgg	
SNA-2 (1-624)F	taaggatccatcgagctc	pGADT7- SNA-2 (1-624)
SNA-2 (1-624)R	agtcatttgtgccaaaatag	
SNA-2 (1-646)F	taaggatccatcgagctc	pGADT7- SNA-2 (1-646)
SNA-2 (1-646)R	tactccggatggattgttatttac	
SNA-2 (602-663)F	tcgtcgttattggatcc	pGADT7- SNA-2 (602-663), pGBKT7- SNA-2 (602-646)
SNA-2 (602-663)R	gaattcactggcctc	
SNA-2 (602-646)F	taaggatccgtcgac	pGBKT7- SNA-2 (602-646)
SNA-2 (602-646)R	tactccggatggattg	
